# Early utilization of hypertonic peritoneal dialysate and subsequent risks of non-traumatic amputation among peritoneal dialysis patients: a nationwide retrospective longitudinal study

**DOI:** 10.1186/1471-2369-14-128

**Published:** 2013-06-20

**Authors:** Shih-Yi Lin, Che-Chen Lin, Chung-Chih Lin, Chi-Jung Chung, Horng-Che Yeh, I-Kuan Wang, I-Wen Ting, Chiu-Chin Huang, Fung-Chang Sung

**Affiliations:** 1 Graduate Institute of Clinical Medical Science, College of Medicine, China Medical University, Taichung, Taiwan; 2 Division of Nephrology and Kidney Institute, China Medical University Hospital, Taichung, Taiwan; 3 Management Office for Health Data, China Medical University Hospital, Taichung, Taiwan; 4 Department of Public Health, China Medical University, Taichung, Taiwan; 5 Department of Medical Research, China Medical University Hospital, Taichung, Taiwan; 6 Department of Health Risk Management and Graduate Institute of Clinical Medical Science, China Medical University, Taichung, Taiwan

**Keywords:** Non-traumatic amputation, Hypertonic dialysate, Peritoneal dialysis

## Abstract

**Background:**

The hemodialysis (HD) population has a particularly high incidence of amputation, which is likely associated with decreased tissue oxygenation during HD. However, information about the risk factors leading to amputation in peritoneal dialysis (PD) patients is limited. Here, we have investigated the association between the use of hypertonic peritoneal dialysate (HPD) and subsequent amputation in PD patients.

**Methods:**

Based on the data from the Taiwan National Health Insurance research database, this observational cohort study enrolled 203 PD patients who had received HPD early during treatment and had not undergone amputation and 296 PD controls who had not undergone amputation. Subjects were followed through until the end of 2009 and the event rates of new non-traumatic amputation were compared between groups.

**Results:**

The incidence of amputation was 3 times higher for the HPD cohort than for the comparison cohort (23.68 vs. 8.01 per 1000 person-years). The hazard ratio (HR) for this group, estimated using a multivariable Cox model, was 2.48 (95% confidence interval [CI] = 1.06–5.79). The HR for patients with both diabetes and early adoption of HPD increased to 44.34 (95% CI = 5.51-357.03), compared to non-HPD non-diabetic PD controls.

**Conclusion:**

Early utilization of HPD in PD patients is associated with increasing risk of amputation; this risk considerably increases for those with concomitant diabetes.

## Background

Non-traumatic extremity amputation, usually related to diabetes, places a considerable burden on individuals, families, and health care finances [1]. Renal failure and dialysis initiation are recognized risk factors for non-traumatic amputation [2,3]. Compared with the general population, dialysis patients experience higher amputation incidence and have much higher post-amputation mortality rates [4]. O’Hare et al reported that hemodialysis (HD) patients who were male, diabetic, had been previously diagnosed with peripheral vascular disease (PVD), had high mean systolic blood pressure, or elevated phosphate levels had increased risks of future amputation [5]. Only a few studies have investigated the risk factors of amputation in peritoneal dialysis (PD) patients, and these have had limited sample size [6,7]. Pliakogiannis et al studied 71 diabetic patients receiving PD and noted that low levels of albumin, peripheral neuropathy, and coronary artery disease were associated with foot lesions [7]. In the clinic, we noted that PD patients who required hypertonic peritoneal dialysis (HPD) solution soon after the initiation of PD were more likely to require amputation. We hypothesize that early HPD use is related to future amputation in PD patients. To test this hypothesis, we conducted a retrospective cohort analysis to assess the incidence of amputation among PD patients and whether this incidence was affected by HPD utilization within the first 6 months of initiating PD.

## Methods

### Data sources

The present study was based on data obtained from the National Health Insurance Research Database (NHIRD) of the National Health Research Institute. The universal National Health Insurance program, launched in March 1995, provides affordable health care to nearly 99% of the population and is contracted with 97% of clinics and hospitals throughout the nation. From the NHIRD, we extracted a Longitudinal Health Insurance Database (LHID), which contained one million beneficiaries, randomly selected from 1996-2000 insurers. The LHID database contains comprehensive annual health care data for all beneficiaries including birth date, gender, diagnostic codes, prescriptions, procedures, and surgeries. The disease diagnosis was coded with International Classification of Diseases, Ninth Revision, Clinical Modification (ICD-9-CM) form outpatient, inpatient and catastrophic illness file. Personal information was de-identified before the release of the research database, thus this study was exempt from approval by the Institutional Review Board.

### Study population

Based on inpatient and outpatient claims, we identified patients who had been first diagnosed with end-stage renal disease (ESRD; ICD-9-CM code 585) and were receiving PD during 1998–2009. Among these new PD patients, subjects prescribed HPD (i.e., 7.5% icodextrin solution or 4.25% dextrose solution) within the first 6 months of initiating PD were identified as the HPD cohort. Those new PD patients who did not received HPD within the first 6 months of initiating PD were considered as the comparison cohort. The index date was the date of initiating PD in the control and in the study cohort. The following patients were excluded: (1) patients who underwent amputation before the index date, (2) patients who experienced severe illness but did not have a record of PD, and (3) patients who had received HD or had undergone renal transplantation before the index date. We followed the cohorts until diagnosis and surgery for the first amputation, either major or minor, (ICD-9-CM 785.4 and 440.24; ICD-9-CM 84.10-84.17) been made, withdrawal from insurance, loss to follow-up, or December 31, 2009, whichever was the latest.

Baseline comorbidities thought to be associated with subsequent amputation were also analyzed. These included diabetes (ICD-9 code 250), hypertension (ICD-9 codes 401-405), ischemic heart disease (ICD-9 codes 410-414, A270, and A279), previous foot ulcers (ICD-9 codes 707.1-707.9), diabetic neuropathy (ICD-9 codes 353.5, 357.2, 354.0-355.9, and 337.1), peripheral vascular disease (PVD, ICD-9-CM codes 443.89 443.9), hyperparathyroidism (ICD-9-CM codes 252.0), heart failure (ICD-9-CM codes 428), diabetes duration, and dialysis vintage.

### Statistical analysis

The differences in demographic characteristics and comorbidities between the study and comparison cohort were examined by using Chi-square test for categorical variables and t-test for continuous variables. We used the Kaplan-Meier method to estimate the amputation-free rates and the log-rank test to examine the statistical significance of the differences between the study groups. Cox proportion hazard model was used to estimate the hazard ratios (HRs) and 95% confidence intervals (CIs) for the risk of subsequent amputation. We further analyzed the interaction between early use of hypertonic dialysate and diabetes. We performed all statistical analysis using SAS 9.1 statistical software (SAS Institute, Inc., Cary, NC, USA) and drew the cumulative incidence curve using R software (R Foundation for Statistical Computing, Vienna, Austria). The differences were considered significant if a two-sided P value was less than or equal to 0.05.

## Results

We identified 499 PD patients eligible for the study, with 203 patients in the HPD cohort and 296 patients in the comparison cohort. The sociodemographic characteristics and comorbidity histories of the 2 groups are shown in Table 1. Compared with the comparison cohort, the HPD cohort was older (mean ages 56.1 vs. 50.9 years, p = 0.0007), and has more prevalence of hypertension (89.7% vs. 83.4%, p = 0.0498), diabetes (43.8% vs. 26.7%, p < 0.0001), diabetes neuropathy (9.4% vs. 4.4%, p = 0.0261) and heart failure (29.6% vs. 20.6%, p = 0.0220). The 2 cohorts did not significantly differ with respect to gender, peripheral vascular disease, and hyperparathyroidism. The mean follow-up duration for the HPD cohort was 3.1 years, which is approximately 1.1 years shorter than that for the comparison cohort.

**Table 1:** Baseline demographic status and comorbidity compared between hypertonic solution cohort and comparison cohort

**Variable**	**PD patients**		**p-value**
	**Comparison cohort**	**HPD cohort**	
	**N = 296 (%)**	**N = 203 (%)**	
Age, mean (SD) years	50.9 (17.1)	56.1 (15.8)	0.0007
≦30	41 (13.9)	12 (5.9)	0.0030
31-50	107 (36.1)	59 (29.1)	
51-70	105 (35.5)	96 (47.3)	
>70	43 (14.5)	36 (17.7)	
Sex			0.3500
Female	170 (57.4)	108 (53.2)	
Male	126 (42.6)	95 (46.8)	
Comorbidity			
Hypertension	247 (83.4)	182 (89.7)	0.0498
Ischemic heart disease	81 (27.4)	70 (34.5)	0.0891
Diabetes	79 (26.7)	89 (43.8)	<0.0001
DM foot ulcer	5 (1.7)	5 (2.5)	0.5445
DM neuropathy	13 (4.4)	19 (9.4)	0.0261
Heart failure	61 (20.6)	60 (29.6)	0.0220
Peripheral vascular disease	5 (1.7)	5 (2.5)	0.5445
Hyperparathyroidism	7 (2.4)	8 (3.9)	0.3111
Follow-up duration, mean (SD)	4.2 (2.9)	3.1 (2.8)	<0.0001
DM duration, mean (SD)	9.8 (3.3)	9.7 (3.6)	0.8736

*t-test.

**Figure 1:**
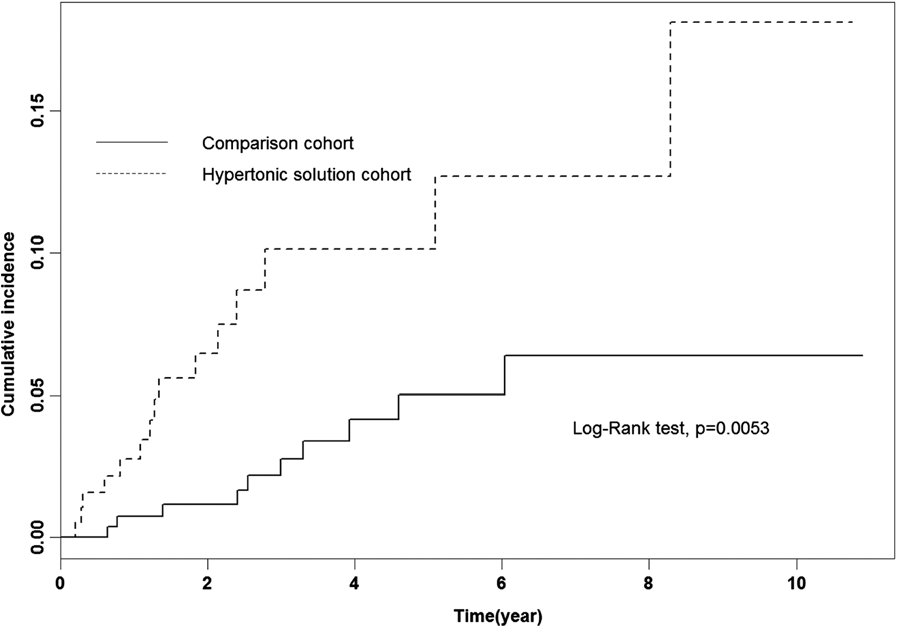
Cumulative incidence of amputation in hypertonic dialysate cohort and comparison cohort.

During the 12-year study period, we identified 25 new amputation patients (15 in the HPD cohort and 10 in the comparison cohort). The incidence density of amputation was higher for the HPD cohort (23.68 per 1000 person-years) than for the comparison cohort (8.01 per 1000 person-years). Thus, the incidence of amputation in the HPD cohort was approximately 3 times higher than that of the comparison cohort. Multivariate regression analysis showed that the risk for new amputation in the HPD cohort was higher than that for the comparison group (HR = 2.48; 95% CI = 1.05–5.79) after adjusting for demographic status, hypertension, ischemic heart disease, and diabetes (Table 2). Further analysis revealed that among patients younger than 70, the rate of amputation for both cohorts increased with age (Table 2). The cumulative incidence of amputation, estimated using the Kaplan-Meier methods showed significant differences and was 0.11-times higher for the HPD cohort than for the comparison cohort (Figure 1, log-rank test p = 0.001). We further analyzed the interaction between diabetes mellitus (DM) and early utilization of HPD (Table 3) and found that diabetic patients had a higher incidence of amputation among all categories, with or without the use of HPD (67.07 and 40.61 per 1000 person-years, respectively). Non-diabetic HPD patients also had a higher incidence of amputation than non-diabetic non-HPD patients (4.55 vs. 0.97 per 1000 person-years), but the risk was not significant (HR = 3.48, 95% CI = 0.31–38.67). For patients who had DM and were receiving HPD, the incidence of amputation increased further to 78.51 per 1000 person-years, with an adjusted HR of 44.34 (95% CI = 5.51–357.03). However, the sample size was not large enough to have the power to detect a significant interaction (p for interaction > 0.05).

**Table 2:** Incidence of amputation and multivariate Cox proportional hazards regression analysis measured hazard ratio for PD patients using hypertonic solution

	**Comparison cohort**			**HPD cohort**				
**Variable**	**Event**	**PY**	**Rate**	**Event**	**PY**	**Rate**	**Crude HR (95% CI)**	**Hazard ratio (95% CI)**
Overall	10	1248	8.01	15	633	23.68	2.98(1.33-6.66)	2.48(1.06-5.79)
Age group								
<50	1	774	1.29	4	311	12.86	10.32(1.15-92.58)	1.20(0.11-12.59)
51-70	8	374	21.35	8	275	29.60	1.41(0.52-3.78)	3.06(0.92-10.22)
>70	1	99	10.08	3	47	63.43	6.24(0.63-61.52)	5.65(0.51-62.54)

*Incidence rate, per 1000 person-years.

*HR* hazard ratio, *CI* confidence interval.

Model adjusted for age, sex, hypertension, ischemic heart disease, heart failure and diabetes.

**Table 3:** The interaction between diabetes and hypertonic solution for amputation risk

**HPD**	**Diabetes**	**Rate**	**Crude HR (95%CI)**	**Adjusted HR (95%CI)**
				
No	No	0.97	ref	ref
No	Yes	40.61	44.32(5.53-355.21)	19.33(2.30-162.12)
Yes	No	4.55	4.80(0.43-53.02)	3.48(0.31-38.67)
Yes	Yes	67.07	78.51(10.3-614.29)	44.34(5.51-357.03)

*Incidence rate, per 1000 person-years.

*HR* hazard ratio, *CI* confidence interval.

Model adjusted for age, sex, hypertension, heart failure and ischemic heart disease.

*p* value for interaction > 0.05.

## Discussion and conclusions

In this nationwide retrospective cohort study, we have illustrated the relationship between early utilization of HPD and subsequent amputation in PD patients. The use of HPD within the first 6 months after initiation of PD was associated with a 2.48-fold increase in the HR of new amputation compared to that for the comparison cohort. Furthermore, HPD cohort who had concomitant DM carried the highest risk, with 44.34 times the incidence of amputation seen in non-HPD non-diabetic patients.

Recent commencement of dialysis therapy has been recognized as an influential risk factor for lower limb amputation in the DM population [2]. Several researchers have studied the pathophysiological changes that occur during hemodialysis, which may contribute to these limb-threatening conditions [8-12]. Systemic hypoxemia, microcirculatory hypoperfusion, and decreased transcutaneous oxygen tension of the lower limbs can occur during and after HD; all these factors could lead to limb ischemia and amputation [9-11]. Although there are no comparable data on PD, it is possible that HPD could produce similar effects. For instance, HPD might create a more rapid fluid shift into the peritoneal cavity, reducing microcirculatory blood flow and tissue oxygen tension. Furthermore, elevated blood pH levels could cause a shift in the oxygen dissociation curve, leading to tissue hypoxia [12]. Furthermore, the use of human recombinant erythropoietin and possible vasoconstriction of microcirculation during ultrafiltration might cause rheological changes and impair microvascular perfusion [6,13]. Moreover, PD patients placed on HPD have clinical signs of fluid overload, such as hypertension and tissue edema, which may worsen tissue oxygenation status. Li et al. found that PD patients have higher incidence rates of mesenteric ischemia than HD patients, indicating that PD therapy might contribute to the advancement of microvascular disease [14]. They hypothesized that chronic exposure to high glucose levels caused micro- and macrovascular damage and contributed to advancing atherosclerosis, which could lead to organ ischemia.

In our study, diabetes was associated with an RR of 19.33 for amputation in PD patients. This result is in accordance with those of previous studies that found that diabetes was the strongest risk factor for amputation in HD patients [5,15-17]. Furthermore, our results showed that diabetic patients tended to initiate HPD earlier than those without diabetes. The risk of amputation with early utilization of HPD and concomitant diabetes was 44.34 times greater than that for PD patients who were neither diabetic nor given HPD therapy. Since diabetics are at greater risk for early ultrafiltration failure, microvasculopathy, and autonomic neuropathy [18-20], early utilization of high-strength dialysate might lead to longer glucose exposure, increased glucose toxicity to the vessels, and finally, limb ischemia, than in non-diabetics. Therefore, we hypothesize that early HPD utilization in diabetic PD patients promotes atherogenesis and/or microvascular hypoperfusion.

Among patients younger than 70, the rate of amputation for both cohorts increased with age. This is likely due to an increased risk of death with surgery for this population, leading to a lower probability of amputation for patients over 70. Eagger et al. found a positive association between increasing age and amputation in the ESRD population [4]. Combes et al. observed that while older age was associated with a decreased probability of amputation in diabetic HD patients, it actually increased the likelihood of amputation among non-diabetics [15]. Pilakogiannis et al reported that age is not related to developing PVD, but no further information is available about the association between age and amputation in PD patients [7]. The inconsistency of these results across studies likely reflects the diverse medical care and mortality rates of different renal populations.

McMurray et al showed that providing diabetic education and care to HD and PD patients significantly improves foot care and reduces the need for amputation [21]. For instance, introduction of the Staged Diabetes Management clinical pathways for dialysis patients have shown to lower the amputation rate considerably [22]. Chiropody been reported could prevent amputation in diabetic PD patients [23]. Thus, amputation in dialysis patients might be preventable in many cases when comprehensive care is provided.

Our study has several strengths. First, it utilized the NHIRD database. This large population database with comprehensive electronic medical records provided complete data about the incidence of amputation as well as important information including that pertaining to age, sex, types of dialysis solution, dialysis vintage, and comorbidities. Second, we excluded subjects with previous amputations and included only PD patients who received long-term follow up care. Excluding patients with past amputation might eliminate possible bias with regard to analyzing future amputation and provide a clearer interpretation of the effect of HPD on limb ischemia.

The several limitations of our study should also be mentioned. First, the number of subjects included was relatively small, leading to large confidence intervals. Furthermore, it is possible that some patients had subclinical PVD. However, we also considered comorbidities such as foot ulcers, heart failure, and cardiovascular risk factors associated with PVD, suggesting that any effect of undiagnosed PVD on our results was likely to be subtle. We also did not have precise information about smoking status and the calcium and phosphate levels, which might be associated with the risk of future amputation. However, Pliakogiannis et al reported that time average Kt/v, creatinine clearance, serum calcium levels, calcium and phosphate production, and intact parathyroid hormone level are not associated with amputation in PD patients [7]. We also lacked information about the subjects’ scores on baseline measures of circulatory status, such as the ankle-brachial index (ABI) [24]. Given that ABI is not correlated with the severity of peripheral arterial disease among dialysis patients and would be falsely elevated by arterial calcification, it may be appropriate to overlook ABI in the current study.

In conclusion, along with diabetes, early utilization of HPD is associated with the subsequent risk of amputation in PD patients. We suggest that PD patients be provided intensive education on foot protection and screening for evidence of limb ischemia, especially those who received HPD early. Further studies are required to determine the efficacy and cost effectiveness of integrated and multidisciplinary foot care in PD patients.

## Competing interests

The authors declare that they have no competing interests.

## Authors’ contributions

SY contributed to every aspect of this article. Both CCL contributed to research data and data analysis. CJ, HC, IK, and CC contributed to study design, discussion, research data, and editing of the manuscript. FC and CCH contributed to the research data, discussion, and review and editing of the manuscript. All authors read and approved the final manuscript.

## Pre-publication history

The pre-publication history for this paper can be accessed here:

http://www.biomedcentral.com/1471-2369/14/128/prepub
